# Blind Persons in Ireland

**Published:** 1861-07

**Authors:** 


BLIND PERSONS IN IRELAND.—It is said that there are 4000 blind women and nearly as many blind men in Ireland. The Irish workhouses contain 1700 blind inmates. It is reported that many of these cases of loss of sight are directly chargeable to a want of personal cleanliness! Dr. Wilde, a high authority on diseases of the eye, declares that the Irish people are more afflicted with blindness than those of any other nation, except the Norwegian.

